# Relationships between primal emotional systems and the sense of loneliness: The mediating role of early maladaptive schemas

**DOI:** 10.1371/journal.pone.0358449

**Published:** 2026-09-15

**Authors:** Monika Talarowska-Dublicka, Aleksandra Gapińska, Maria Finogenow

**Affiliations:** Institute of Psychology, Faculty of Educational Sciences, University of Lodz, Poland; Sapienza University of Rome: Universita degli Studi di Roma La Sapienza, ITALY

## Abstract

**Background:**

Within Panksepp’s neuroaffective theory, emotional–motivational systems constitute the biological basis for individual differences in the experience and regulation of emotions, which may be related to loneliness. At the same time, these systems may be associated with the formation of early maladaptive schemas which – according to Young’s concept – affect interpersonal functioning. The aim of this study was to examine the relationships between emotional–motivational systems and the sense of loneliness, and to determine whether early maladaptive schemas statistically mediate these relationships.

**Materials and methods:**

The study involved 323 participants aged 18 to 66 years (mean age: M = 26.9). The following instruments were used: the Revised UCLA Loneliness Scale (R-UCLA), the Brief Form of the Affective Neuroscience Personality Scales (BANPS), and the Young Schema Questionnaire – Short Form (YSQ-S3-PL).

**Results:**

All maladaptive schemas, schema domains, and systems – except for the SEEKING system – were significantly associated with the sense of loneliness. The strongest associations were observed for the SADNESS / PANIC / SEPARATION DISTRESS and FEAR systems. Emotional–motivational systems were also associated with early maladaptive schemas, with the strongest relationships found for four systems: SADNESS / PANIC / SEPARATION DISTRESS, FEAR, RAGE, and PLAY. Schema domains showed significant statistical mediation in the relationships between primal emotional systems and loneliness, except for the SEEKING system, where no mediation effect was found. The primary mediator in the tested relationships was the Disconnection/Rejection domain.

**Conclusions:**

The findings suggest that early maladaptive schemas related to unmet needs for belonging, love, and relational safety are associated with the relationships between primal emotional systems and loneliness. In particular, the Disconnection/Rejection schema domain emerged as the most prominent statistical mediator in the analyzed models.

## Introduction

The phenomenon of loneliness experienced by contemporary individuals has been receiving increasing attention in recent years [1]. It is, however, important to distinguish between loneliness – characterized by a negative affective tone – and solitude, which represents a conscious personal choice. In the literature, loneliness is defined as the subjective experience of lacking satisfying social relationships. It does not necessarily coincide with an actual (objective) absence of social bonds [2]. Solitude, by contrast, is a voluntary choice accompanied by a neutral or positive mood, often aimed at enhancing self-awareness, seeking rest, or even fostering creativity [3]. It is also regarded as one of the more effective strategies for reducing internal tension and discomfort [4].

The detrimental effects of prolonged loneliness on both physical and mental health are now well established [5]. Numerous large-scale meta-analyses have confirmed associations between perceived social isolation and an increased risk of cardiovascular disease [6,7], metabolic disorders [8], cancer [9], depressive disorders [10], and dementia [11,12]. Explanations for these associations have been sought in elevated cortisol levels characteristic of prolonged distress accompanying social isolation [13,14], pathological activity of the hypothalamic–pituitary–adrenocortical (HPA) axis [15], as well as oxidative stress and heightened inflammatory processes [16].

In our view, there remains a paucity of studies that integrate biological variables with diverse environmental factors contributing to social isolation, particularly when examined from a developmental perspective. Such a perspective could provide important insights into interindividual differences in the intensity of loneliness experienced. Taking this developmental aspect into account directs attention toward two potentially interconnected theoretical frameworks: Jaak Panksepp’s Affective Neuroscience Theory (ANT), which addresses the biological basis of loneliness [17], and Jeffrey Young’s theory of Early Maladaptive Schemas (EMS), which encompasses cognitive, environmental, relational, and developmental aspects.

Panksepp’s theory identifies six emotional–motivational systems (networks of brain centers and their connections – neural circuits – that play a crucial role in basic emotional processes and their corresponding behaviors), located in the evolutionarily older, subcortical structures of the mammalian brain (both in humans and other animals) [18]. These systems are responsible for the activation and coordination of responses to stimuli from both the external and internal environment [19].

The emotional systems identified by Panksepp are: SEEKING (interest), RAGE (anger), FEAR (anxiety), CARE (nurturance), SADNESS / PANIC / SEPARATION DISTRESS (grief), and PLAY (playfulness / joy) [20]. Each system is activated through stimulation of distinct – though partially overlapping – brain regions. Typically, they operate in concert to enhance the adaptiveness of an individual’s feelings, thoughts, and behaviors [21].

Of these systems, the following appear to be of particular relevance to loneliness: SADNESS / PANIC / SEPARATION DISTRESS, SEEKING, CARE, and PLAY. The first of these is strongly activated when an individual experiences an actual loss of social relationships or feels abandoned or isolated. Its activation is linked to emotions such as fear of losing an attachment figure and the suffering that follows such loss. Anatomically, this system is associated with the anterior cingulate cortex, bed nucleus of the stria terminalis (BNST), dorsomedial hypothalamus, preoptic area, ventral tegmental area (VTA), and periaqueductal gray matter (PAG) [22]. The neurotransmitters and neuromodulators involved in this system include opioids, prolactin, oxytocin, corticotropin-releasing hormone, and glutamic acid. According to Bonanno et al. [23], prolonged activation of the SADNESS / PANIC / SEPARATION DISTRESS system may be associated with chronic negative emotionality, transforming normal sadness into a clinical disorder associated with loneliness, social withdrawal, despair, avoidance of activity, persistent negative thoughts, and behavioral disturbances [23]. Davis and Montag [24] directly linked the experience of loneliness to activation of the neural circuits underlying this system. Overactivity of the SADNESS / PANIC / SEPARATION DISTRESS system during childhood – resulting from a persistent absence of close, stable bonds – may correspond to lasting changes in the limbic system, which in adulthood manifest as, on the one hand, heightened sensitivity to rejection and, on the other, fear of rejection in social relationships [25].

The second emotional system potentially relevant to the development of heightened loneliness is the SEEKING system (interest, curiosity, search for positive reinforcement). This system is responsible, among other functions, for generating motivation to explore the environment in conjunction with forming social connections. According to Panksepp, it is the primary and evolutionarily oldest emotional system among all affective systems [26]. Activation of the SEEKING system initiates intense learning processes – producing adaptive behaviors (basal ganglia) and knowledge (neocortex). It is associated with activity in structures including the nucleus accumbens, ventral tegmental area, and lateral hypothalamus–periaqueductal gray matter (PAG), as well as mesolimbic and mesocortical pathways [18]. The functioning of the SEEKING system is interlinked with the SADNESS / PANIC / SEPARATION DISTRESS system – when efforts to build satisfying social relationships prove unsuccessful, the former system is “switched off” (resulting in reduced motivation to initiate prosocial actions and decreased satisfaction with their occurrence) [27]. This process is a consequence of prolonged stress and ultimately manifests as behavioral withdrawal. Another effect of activating this interrelated network is stimulation of the RAGE system, whose operation is expressed in increased feelings of anger or rage and in aggressive behaviors directed toward the social environment [28].

Another system of significant importance for the development of loneliness is the CARE system. It governs responses associated with maternal and caregiving behaviors and with the formation of interpersonal bonds. This system is responsible for creating and maintaining social ties, as well as for nurturing and attachment. Proper functioning of this system is crucial for achieving and sustaining a sense of safety and belonging, whereas its dysfunction is strongly associated with the development and maintenance of loneliness [24]. It is particularly active during the early developmental period of childhood, with its functioning linked to the activation of opioid systems in the brain as well as the oxytocin and prolactin systems. The key anatomical regions of the human brain involved in this system include the anterior cingulate cortex, bed nucleus of the stria terminalis, preoptic area, ventral tegmental area, and periaqueductal gray matter (PAG) [29]. Specific early childhood experiences involving profound emotional deprivation, through suppression of CARE system activity, may be associated with higher levels of loneliness in adulthood [30].

The final neuroaffective system of potential relevance to the intensity of loneliness is the PLAY system. It is responsible for prosocial behaviors, adaptation to social norms and patterns, and the acquisition of social competence. Although it does not have direct adaptive significance for survival, it plays an important role in building social bonds. Its activation may be associated with lower levels of loneliness [31]. Emotionally, it is associated with the reduction of negative affect and modulates the functioning of other emotional systems. It is regulated primarily by dopamine, oxytocin, and cannabinoids. The brain regions characteristic of its activity include the dorsomedial midbrain, periaqueductal gray matter, posterior dorsomedial thalamic nuclei, and the parafascicular thalamic nucleus [19].

The remaining two emotional systems identified in Panksepp’s theory – FEAR and RAGE – do not appear, according to the literature, to be directly involved in the experience of loneliness; however, they may influence its intensity.

The FEAR system is engaged in continuous monitoring of the environment, enabling threat avoidance. It is responsible for initiating escape and/or freezing responses and is regulated primarily by the serotonergic system. Emotionally, it is associated with feelings of fear and anxiety. The main brain regions linked to its functioning are the amygdala, anterior and medial hypothalamus, and periaqueductal gray matter [24]. Activation of this system may enhance avoidance and withdrawal tendencies, which, through social isolation, can in turn intensify the experience of loneliness [31].

The RAGE system was previously mentioned in relation to the SEEKING system. Emotionally, it is associated with feelings of anger and rage. Its activity is regulated by testosterone, substance P, noradrenaline, glutamate, acetylcholine, and gamma-aminobutyric acid (GABA). The main brain regions characteristic of its activation include the amygdala, hypothalamus, stria terminalis, and periaqueductal gray matter [32]. Excessive or uncontrolled activation of this system may be associated with more frequent social conflicts and greater social isolation, both of which may be linked to higher levels of loneliness [31].

In Panksepp’s affective neuroscience framework, emotions are innate regulatory mechanisms, and the associated neural circuits ensure the most effective adaptation to environmental demands. According to Panksepp [33], emotions constitute a fundamental basis of personality, and personality assessment may provide insight into these subcortical affects. Moreover, differences in the reactivity of primary emotional systems may be understood as the foundation of individual differences in personality [29].

In Paul Ekman’s model of basic emotions, as integrated into Young’s early maladaptive schema theory, the activation of basic emotions is related to the satisfaction or frustration of early childhood needs. Attachment needs, when frustrated, typically evoke fear and sadness. Disgust or anger indicates frustration of the need for control, independence, or assertiveness. In contrast, joy signifies a satisfactory level of need fulfilment, regardless of its source [34]. According to Young’s theory, failure to meet core emotional needs – such as unconditional acceptance, closeness, and attachment; autonomy and identity; realistic limits; spontaneity and play; and free expression of emotions and needs – is associated in early childhood with the development of maladaptive coping styles in response to intense emotions resulting from these frustrations. These maladaptive coping styles persist into adulthood, contributing to a broad range of psychiatric symptoms [35]. From a neurobiological perspective, the formation of early maladaptive schemas is rooted in the primal emotional systems and their connections with the frontal cortex and limbic system, while from an environmental perspective, it is related to the nature of social experiences and the degree to which basic emotional needs are fulfilled [36]. Furthermore, Ekman and Cordaro [37] suggest that humans possess an “open affective program”, which is susceptible to and enriched by new information derived from experience. Once new data are integrated into the program, it functions automatically in accordance with biological predispositions [37]. These assumptions again allow for linking the neural basis of affective systems with the environmental experiences that shape early maladaptive schemas.

Using a developmental perspective, the present study seeks to explore the biological underpinnings of loneliness, assessing the mediating role of early maladaptive schemas as a factor that may significantly influence the degree of discomfort experienced as a consequence of relational distress. Detailed analyses of the connections between early maladaptive schemas and emotional systems have been described in our previous work [38,39].

Based on the findings of our previous research [38,39], we hypothesize that the early maladaptive schema domain Disconnection/Rejection – associated with the unmet need for unconditional acceptance, closeness, and attachment – is the strongest mediator between primal emotional systems (particularly SADNESS / PANIC / SEPARATION DISTRESS, SEEKING, CARE, and PLAY) and the intensity of loneliness.

## Materials and methods

The study included 323 participants aged 18–66 years (M = 26.9; SD = 10.7), of whom 56% (N = 180) were women, 43% (N = 140) were men, and 1% (N = 3) did not declare either option.

The study was conducted remotely using the Microsoft Forms platform. Participants were recruited by students of the Institute of Psychology at the University of Lodz, Poland, between 1^st^ March 2023 and 31^st^ January 2024 through announcements posted in publicly accessible social media groups. Inclusion criteria were: age over 18 years, no history of diagnosed mental disorders (participants who declared current or past treatment for mental disorders were not allowed to complete the remaining parts of the study and were excluded from further participation), and provision of informed consent to participate in the study. The sociodemographic survey included a question regarding past and current psychiatric treatment history. Individuals who responded affirmatively to this question were excluded from the study. The characteristics of the study group are presented in Table 1.

**Table 1:** Characteristics of the study group by age, place of residence, and education.

Quantitative variables	Min	Max	M	SD
Age	18	66	26.9	10.7
Qualitative variables			N	%
Place of residence	Rural area		59	18.3%
	Town ≤ 100,000 inhabitants		66	20.4%
	City > 100,000 inhabitants		198	61.3%
Education	Primary/lower secondary		4	1.2%
	Vocational		4	1.2%
	Secondary		180	55.7%
	Higher		135	41.8%
Marital status	Married Single Cohabiting/partnered Divorced Separated Widowed		38 180 94 10 – 1	11.8% 55.7% 29.1% 3.0% – 0.3%

Min, minimum; Max, maximum; M, mean; SD, standard deviation; N, number of participants; %, percentage of the subgroup within the total study sample.

The study protocol was approved by the Research Ethics Committee of the Medical University of Lodz (decision no. RNN/92/22/KE, dated 14 June 2022).

Only adults participated in the study. Each participant provided informed written consent to participate in the study.

After providing informed consent, each participant completed an online survey consisting of a demographic questionnaire, the R-UCLA scale, the BANPS questionnaire, and the YSQ-S3-PL questionnaire.

Instruments used in the study:

a. Revised UCLA Loneliness Scale (R-UCLA)

The Revised UCLA Loneliness Scale [40] is a tool used to assess loneliness as a trait. It demonstrates high theoretical validity and reliability [41]. The instrument consists of 20 items, grouped into three factors (subscales). The first subscale, Intimate Others, contains ten items assessing the respondent’s feelings in relation to connection with a group or isolation from others. Higher scores indicate a stronger sense of loneliness expressed as a lack of close relationships and separation from others. The second subscale, Social Others, consists of five items reflecting loneliness in terms of social contact with people in the immediate environment or the group as a whole. High scores indicate the absence of people with whom the respondent feels closely connected, who truly understand them, and the lack of a social network or closeness in relationships. The third subscale, Belonging and Affiliation, consists of five items measuring integration with and belonging to a group, referring to the absence of group identity and community bonds, weaker social ties, and the perception of oneself more as an individual than as part of a group.

Given the complexity of the analyses and the number of variables in the present study, the total score from all three subscales was used in the analyses as an indicator of global loneliness (the higher the score, the greater the sense of loneliness). In the current sample, Cronbach’s α was .92.

b. Brief Form of the Affective Neuroscience Personality Scales (BANPS)

Originally developed by Barrett et al. [42], this method measures the intensity of activity in each of the six neuroaffective emotional systems based on the respondent’s self-report. The BANPS assesses personality traits reflecting the functional expression of underlying neurobiological emotional systems, rather than direct neural activation [42]. Therefore, the terminology used in our manuscript refers to the theoretical neurobiological constructs described in affective neuroscience rather than to directly measured neural activity. This approach is consistent with previous research using ANPS/BANPS, where the constructs are described as primary emotional systems rooted in subcortical neural circuitry but measured through behavioral or personality indicators [24,32].

Participants rate their agreement with 33 statements on a Likert scale ranging from 1 (strongly disagree) to 5 (strongly agree). The final score for each of the six BANPS subscales is the sum of the scores assigned by the respondent to the items included in that subscale. The English version of the BANPS is available in open access [42]. The Polish adaptation of the scale was developed by the authors of the present article. A translation method was applied, allowing for minor modifications where literal translation was not possible while maintaining semantic equivalence [43].

In the first step, following established standards, the original BANPS was translated into Polish by three independent psychologists fluent in English and with theoretical and practical expertise in personality theory. Comparison of the translations revealed discrepancies in four items (i.e., 8, 17, 21, and 24), mainly due to synonym usage or challenges in preserving both translation accuracy and semantic equivalence.

Through group discussion, the most optimal wording for the majority of items was determined. Next, a native Polish speaker with a background in psychology and fluent English proficiency was asked to perform a blind back-translation. Any additional discrepancies identified at this stage were resolved through discussion among the initial translators.

Finally, a pilot study was conducted with 50 psychology students from the University of Lodz, who were invited to provide feedback regarding the structure of the questionnaire and the clarity of individual items.

The mean completion time was 25 minutes. This process confirmed the adequacy of the official Polish version of the questionnaire, which was subsequently subjected to psychometric evaluation. In the Polish BANPS version, the following subscale names were adopted: *ZABAWA*, *GNIEW*, *CIEKAWOŚĆ*, *OPIEKUŃ*CZO*ŚĆ*, *SMUTEK*, *STRACH* (corresponding to PLAY, RAGE, SEEKING, CARE, SADNESS, and FEAR). The Polish version demonstrates psychometric properties comparable to other language adaptations [44]. In the current sample, Cronbach’s α values were .79, .77, .70, .53, .87, and .75, respectively.

c. Young Schema Questionnaire – Short Form (YSQ-S3-PL)

Originally developed by Young et al. [45], the Young Schema Questionnaire – Short Form (YSQ-S3) measures the intensity of 18 schemas based on the respondent’s self-report. Participants rate their agreement with 90 statements on a Likert scale from 1 (completely untrue of me) to 6 (describes me perfectly). The final score for each of the 18 subscales, which reflect the intensity of individual schemas, as well as the higher-order scores representing schema domains, enables the identification of a schema profile characteristic for a given individual. The present study used the Polish adaptation of the scale [46]. In the current sample, Cronbach’s α coefficients ranged from .63 (Entitlement) and .74 (Unrelenting Standards) to .91 (Failure) and .92 (Defectiveness). For the schema domains, Cronbach’s α coefficients ranged from .78 (Impaired Limits) to .95 (Disconnection and Rejection).

### Statistical methods

The Statistical Package for the Social Sciences (SPSS), version 29.0, was used to calculate descriptive statistics, perform correlation analyses, and conduct regression analyses for mediation testing.

For nominal variables, the frequency of occurrence in the sample was presented as an absolute value (number of observations, N) and as a percentage (%). Continuous variables were expressed as means with standard deviations. To evaluate the reliability of the scales, Cronbach’s α coefficients were computed for each subscale.

The normality of distribution for continuous variables was assessed using the Shapiro–Wilk W test, histogram analysis, skewness, and kurtosis. Because Shapiro–Wilk tests are highly sensitive in larger samples, particular emphasis was placed on skewness and kurtosis values as indicators of practical distribution normality. All skewness and kurtosis values were within acceptable ranges for the application of parametric statistical methods. For all analyses, the significance level was set at p ≤ 0.05.

Due to the confirmed normal distribution of the studied variables, parametric statistical methods were applied. To examine the relationships between variables, as well as their direction and strength, Pearson’s r correlation coefficient was used. Mediation analysis was conducted using the bootstrapping procedure described by Preacher and Hayes [47], which enables estimation of indirect effects without assuming the normality of distribution. This assumption is required in the classical mediation approach proposed by Baron and Kenny [48] and in the Sobel test, but it is often not met in practice. Bootstrapping circumvents this limitation. In the present study, Model 4 of the PROCESS macro (version 4.2) by Hayes [49] was used, with 10,000 bootstrap samples to compute 95% bias-corrected confidence intervals for the estimation of indirect effects. An indirect effect was considered statistically significant if its confidence interval did not include zero [49]. The use of a parallel mediation model allowed identification of which mediators accounted for the mediation effect, as well as comparison of the strength and importance of specific indirect effects [50]. The analysis estimated the total effect, direct effect, total indirect effect, and specific indirect effects for each mediator. All EMS domains were simultaneously entered into a single model as mediators, which enabled examination of their mediating role in the relationship between emotional systems and loneliness.

## Results

A summary of the results obtained from the individual questionnaires (R-UCLA, BANPS, and YSQ-S3-PL) is presented in Table 2 (N = 323).

**Table 2:** Summary of results obtained in the R-UCLA, BANPS, and YSQ-S3-PL questionnaires (N = 323).

Variable	Min	Max	M	SD	Skewness	Kurtosis
**R-UCLA (Total score)**	21	75	44.04	11.88	.252	−.418
**SADNESS / PAIN / SEPARATION DISTRESS (BANPS)**	6	30	19.36	5.84	−.142	−.633
**SEEKING (BANPS)**	8	30	21.55	4.18	−.151	.129
**CARE (BANPS)**	4	20	14.35	3.11	−.324	−.124
**PLAY (BANPS)**	6	30	21.19	4.65	−.570	−.016
**FEAR (BANPS)**	6	25	18.04	4.37	−.274	−.539
**RAGE (BANPS)**	6	30	17.25	5.32	.246	−.497
**Rejection / Disconnection (YSQ-S3-PL)**	**25**	**149**	**71.26**	**27.16**	**.456**	**−.471**
**Emotional deprivation**	5	30	12.31	6.13	.774	−.021
**Abandonment/instability**	5	30	16.21	6.96	.290	−1.027
**Mistrust/abuse**	5	30	15.02	6.77	.361	−.881
**Social isolation/alienation**	5	30	15.89	6.62	.223	−.876
**Defectiveness/shame**	5	30	11.85	6.94	.945	−.126
**Impaired Autonomy and Performance (YSQ-S3-PL)**	**20**	**114**	**50.79**	**20.39**	**.621**	**−.219**
**Failure to achieve**	5	30	14.21	7.04	.464	−.882
**Dependence/incompetence**	5	29	12.46	5.90	.756	−.088
**Vulnerability to harm or illness**	5	30	13.46	6.47	.672	−.315
**Enmeshment/undeveloped self**	5	30	10.67	5.27	1.161	1.072
**Other-directedness (YSQ-S3-PL)**	**15**	**83**	**46.76**	**12.82**	**.205**	**0.12**
**Subjugation**	5	30	13.52	5.90	.527	−.506
**Self-sacrifice**	5	29	15.91	4.69	.246	−.277
**Approval seeking/recognition seeking**	5	30	17.34	5.83	.108	−.602
**Impaired Limits (YSQ-S3-PL)**	**10**	**55**	**31.74**	**8.71**	**−.096**	**−.083**
**Entitlement/grandiosity**	5	30	14.89	4.54	.188	.068
**Insufficient self-control/self-discipline**	5	30	16.86	6.20	.118	−.804
**Overvigilance and Inhibition (YSQ-S3-PL)**	**20**	**111**	**61.85**	**19.16**	**.161**	**−.525**
**Negativity/pessimism**	5	30	16.31	6.66	.217	−.899
**Emotional inhibition**	5	30	14.98	6.06	.313	−.659
**Unrelenting standards/Hypercriticalness**	5	30	17.16	5.60	.047	−.704
**Punitiveness**	5	30	13.41	5.84	.640	−.025

R-UCLA, Revised UCLA Loneliness Scale; BANPS, Brief Form of the Affective Neuroscience Personality Scales; YSQ-S3-PL, Young Schema Questionnaire, Short Form, Polish version; Min, minimum; Max, maximum;M, mean; SD, standard deviation.

### Relationships between primal emotional systems, early maladaptive schemas, and the sense of loneliness

To examine the relationships between emotional systems, early maladaptive schemas, and the sense of loneliness, Pearson’s *r* correlation analyses were conducted. The analysis included all 18 maladaptive schemas as well as the five schema domains.

The results showed that all maladaptive schemas and schema domains were significantly and positively associated with the sense of loneliness (see Table 3). Emotional systems – except for the SEEKING system – were also significantly associated with loneliness.

**Table 3:** Relationships between emotional systems, early maladaptive schemas, and the sense of loneliness*.*

	Loneliness	SADNESS	SEEKING	CARE	FEAR	RAGE	PLAY
**Loneliness**		.55***	−.09	−.23***	,25***	.19***	−.43***
**Abandonment/instability**	.49***	.59***	−.03	.16**	.56***	.30***	−.20***
**Mistrust/abuse**	.60***	.39***	.03	−.13*	.36***	.28***	−.28***
**Emotional deprivation**	.72***	.41***	−.13*	−.22***	.13*	.19***	−.26***
**Defectiveness/shame**	.61***	.47***	−.14**	−.16**	.29***	.28***	−.27***
**Social isolation/alienation**	.74***	.48***	.01	−.18**	.29***	.19***	−.40***
**Dependence/incompetence**	.51***	.49***	−.20***	.06	.48***	.26***	−.26***
**Vulnerability to harm or illness**	.45***	.43***	−.04	.02	.56***	.22***	−.29***
**Enmeshment/undeveloped self**	.35***	.31***	−.14*	.03	.39***	.15**	−.15**
**Failure to achieve**	.46***	.40***	−.25***	−.01	.43***	.19***	−.20***
**Entitlement/grandiosity**	.19***	.09	.16**	−.11*	.02	.24***	−.02
**Insufficient self-control/discipline**	.27***	.31***	−.09	.04	.34***	.28***	−.04
**Subjugation**	.55***	.46***	−.16**	.02	.44***	.13*	−.30***
**Self-sacrifice**	.22***	.17**	.12*	.15**	.13*	<.01	−.06
**Approval seeking/recognition seeking**	.24***	.33***	.07	.09	.40***	.28***	−.06
**Negativity/pessimism**	.51***	.50***	−.07	.02	.60***	.25***	−.30***
**Emotional inhibition**	.55***	.33***	−.07	−.32***	.26***	.01	−.44***
**Unrelenting standards**	.29***	.30***	.14*	−.01	.36***	.04	−.20***
**Punitiveness**	.48***	.35***	−.07	−.06	.25***	.16**	−.27***
**SD. Disconnection and rejection**	.77***	.58***	−.06	−.12*	.41***	.30***	−.35***
**SD. Impaired autonomy**	.54***	.50***	−.19***	.03	.57***	.25***	−.28***
**SD. Impaired limits**	.29***	.27***	.02	−.03	.25***	.32***	−.04
**SD. Other-directedness**	.44***	.43***	<.01	.11	.43***	.19***	−.19***
**SD. Overvigilance/inhibition**	.58***	.47***	−.03	−.12*	.47***	.15**	−.38***

SD, Schema Domain; *, p statistically significant; *p ≤ .05; **p ≤ .01; ***p ≤ .001.

The strongest associations, as well as the highest number of significant relationships between primal emotional systems and early maladaptive schemas, were observed for the SADNESS / PANIC / SEPARATION DISTRESS and FEAR systems. These systems were significantly associated with all schema domains and with the majority of schemas, with the exception of the Entitlement/grandiosity schema. The RAGE system was significantly associated with all schema domains and with most schemas, except for the Self-sacrifice, Emotional inhibition, and Unrelenting standards schemas. The PLAY system was significantly associated with most schema domains, except for the Impaired Limits domain, and with most schemas, except for Entitlement/grandiosity, Insufficient self-control/self-discipline, Self-sacrifice, and Approval seeking/recognition seeking. The SEEKING system was significantly and negatively related to one schema domain – Impaired Autonomy and Performance – and to the schemas of Emotional deprivation, Defectiveness/shame, Dependence/incompetence, Enmeshment, Failure to achieve, and Subjugation, but significantly and positively associated with the Entitlement/grandiosity and Unrelenting standards schemas. The CARE system was significantly and negatively related to the Disconnection/Rejection and Overvigilance and Inhibition domains, as well as to the Mistrust/abuse, Emotional deprivation, Defectiveness/shame, Social isolation/alienation, Entitlement/grandiosity, and Emotional inhibition schemas, but significantly and positively associated with the Abandonment/instability and Self-sacrifice schemas.

In summary, the sense of loneliness and affective systems are significantly related to the intensity of specific early maladaptive schemas and their domains. However, differences are observed in the strength and direction of these relationships.

### The mediating role of early maladaptive schemas in the relationship between emotional systems and the sense of loneliness

**Fig 1:**
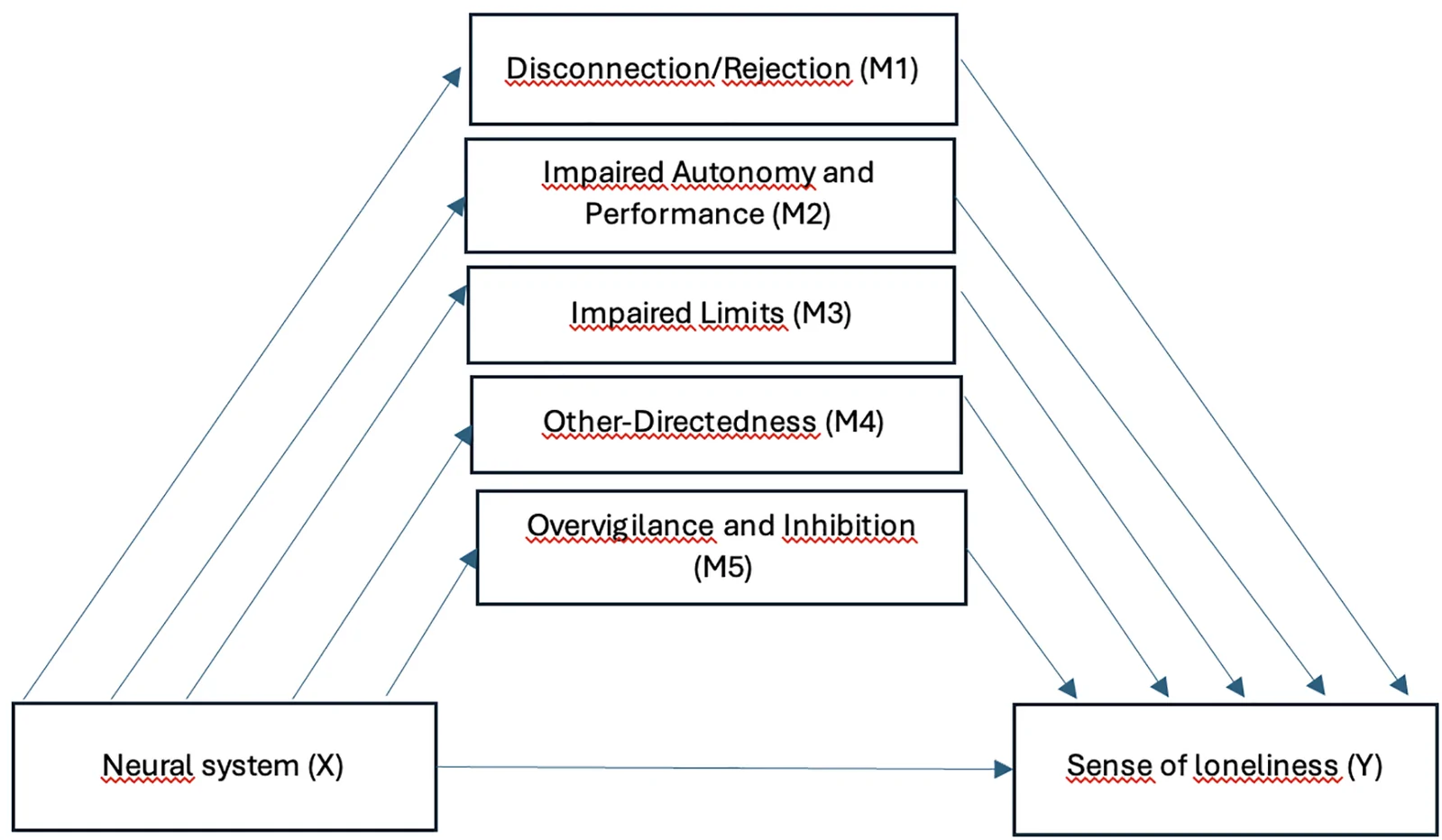
Model of the relationship between affective systems, schema domains, and the sense of loneliness. Source: author’s own work.

Next, we examined whether schema domains mediate the relationship between affective systems and the sense of loneliness. To this end, parallel mediation analyses were conducted using the PROCESS macro (Model 4; Hayes, 2022), applying bootstrapping with 10,000 samples. Emotional systems were entered as independent variables (X), schema domains as mediators (M1, M2, M3, M4, M5), and the sense of loneliness as the dependent variable (Y). The conceptual model is presented in Fig 1, and a summary of the analyses is shown in Table 4.

**Table 4:** Summary of the relationships between emotional systems, schema domains, and the sense of loneliness.

Emotional system	Total Effect (β)	Direct Effect (β)	Total Indirect Effect (β)	Significant Mediators	Mediation Type
**SADNESS/ PANIC/ SEPARATION DISTRESS**	.55 (p < .001)***	.17 (p < .001)***	.38 (p < .001)***	**M1**	Partial
**FEAR**	.25 (p < .001)***	−.06 (p = .191)	.31 (p < .001)***	**M1, M3**	Full
**RAGE**	.19 (p < .001)***	−.04 (p = .358)	.22 (p < .001)***	**M1**	Full
**CARE**	−.23 (p < .001)***	−.14 (p < .001)***	−.10 (p < .05)*	**M1**	Partial
**PLAY**	−.43 (p < .001)***	−.19 (p < .001)***	−.24 (p < .001)***	**M1**	Partial
**SEEKING**	−.09 (p = .097)	−.05 (p = .136)	−.04 (n.i)	None	–

M1, Disconnection/Rejection; M3, Impaired Limits; *p ≤ .05; ***p ≤ .001.

In the first analysis, the **SADNESS / PANIC / SEPARATION DISTRESS** system was entered as the independent variable (X), the sense of loneliness as the dependent variable (Y), and the schema domains as mediators. Results indicated that the associations between SADNESS / PANIC / SEPARATION DISTRESS (X) and all mediators were statistically significant and positive (M1: β = .58; p < .001; M2: β = .50; p < .001; M3: β = .27; p < .001; M4: β = .43; p < .001; M5: β = .47; p < .001). Among the mediators, only the Disconnection/Rejection domain (M1) was significantly related to loneliness (β = .80, p < .001). The total effect of SADNESS / PANIC / SEPARATION DISTRESS on loneliness was significant (β = .55, SE = .14, 95% CI [1.41, 1.97]). The direct effect also remained significant after including the mediators, although smaller (β = .17, SE = .13, 95% CI [.26, .78]), indicating partial mediation. The total indirect effect was significant (β = .38, BootSE = .04, 95% CI [.31, .45]), with the only significant indirect pathway running through Disconnection/Rejection (β = .46, BootSE = .04, 95% CI [.38, .55]). In contrast, the indirect effects via M2 (β = −.05, BootSE = .03, 95% CI [−.11, .01]), M3 (β = −.02, BootSE = .01, 95% CI [−.05, .01]), M4 (β = −.02, BootSE = .02, 95% CI [−.07, .03]), and M5 (β = .01, BootSE = .03, 95% CI [−.05, .07]) were not significant and very small.

In the second analysis, the **SEEKING** system was treated as the independent variable. SEEKING **significantly predicted only one of the five schema domains – Impaired Autonomy and Performance (M2)** – and the **association was negative** (β = −.19, p < .001). No significant relationships were found with other domains. Of the mediators, only Disconnection/Rejection (M1) significantly predicted loneliness (β = .88, p < .001). The remaining domains were not significantly associated with loneliness. The total effect of SEEKING on loneliness **was not significant** (β = −.09, SE = .16, 95% CI [−.60, .05]), nor was the direct effect after controlling for mediators (β = −.05, SE = .11, 95% CI [−.38, .05]). The total indirect effect was also nonsignificant (β = −.04, BootSE = .04, 95% CI [−.12, .05]), and **none of the indirect effects reached significance**: M1 (β = −.06, BootSE = .05, 95% CI [−.15, .04]), M2 (β = .02, BootSE = .01, 95% CI [−.01, .05]), M3 (β < −.01, BootSE = .01, 95% CI [−.01, .01]), M4 (β < .01, BootSE < .01, 95% CI [−.01, .01]), and M5 (β < .01, BootSE = .01, 95% CI [−.01, .01]).

In the third analysis, the **CARE** system was entered as the independent variable. CARE (X) was significantly and **negatively** related to two mediators: Disconnection/Rejection (M1) (β = −.12, p < .05) and Overvigilance and Inhibition (M5) (β = −.12, p < .05). The association with the Impaired Limits domain (M4) was at the threshold of significance (β = .11, p = .05). The remaining associations were nonsignificant (M2: β = .03, p > .05; M3: β = −.03, p > .05). Among the mediators, Disconnection/Rejection (M1) (β = .84, p < .001) and Impaired Limits (M3) (β = −.11, p < .05) significantly predicted loneliness. The remaining domains were not significantly associated with the sense of loneliness. The **total effect** of CARE on loneliness was significant and negative (β = −.23, SE = .22, 95% CI [–1.35, −.50]). The **direct effect** also remained significant (β = −.14, SE = .15, 95% CI [−.84, −.26]), indicating **partial mediation**. The **total indirect effect** was significant (β = −.10, BootSE = .05, 95% CI [−.18, −.00]), with the only **significant** (but small) **indirect effect** occurring through **Disconnection/Rejection** (M1) (β = −.10, BootSE = .05, 95% CI [−.20, −.01]). The indirect effects via M2 (β < −.01, BootSE < .01, 95% CI [−.01, .01]), M3 (β < .01, BootSE = .01, 95% CI [−.01, .02]), M4 (β < .01, BootSE = .01, 95% CI [−.01, .02]), and M5 (β < .01, BootSE = .01, 95% CI [−.01, .02]) were not significant and very small.

In the fourth analysis, the **FEAR** system was the independent variable. **FEAR (X)** significantly predicted all five schema domains (**M1**: β = .41, p < .001; **M2**: β = .57, p < .001; **M3**: β = .25, p < .001; **M4**: β = .43, p < .001; **M5**: β = .47, p < .001). Among the mediators, significant predictors of loneliness (Y) were Disconnection/Rejection (**M1**) (β = .87, p < .001) and, **negatively**, Impaired Limits (**M3**) (β = −.09, p = .049). The remaining domains were not significantly associated with loneliness. The **total effect** of the FEAR system on loneliness was significant and positive (β = .25, SE = .15, 95% CI [.41, 1.01]). However, after accounting for the mediators, the **direct effect** became nonsignificant (β = −.06, SE = .12, 95% CI [−.40, .08]), suggesting **full mediation**. The **total indirect effect** was significant (β = .31, BootSE = .05, 95% CI [.21, .40]). Significant indirect effects were observed for two domains: Disconnection/Rejection (**M1**) (β = .36, BootSE = .05, 95% CI [.26, .45]) and Impaired Limits (**M3**) (β = −.02, BootSE = .01, 95% CI [−.05, < −.01]) – however the second one was significant but very small. The remaining indirect effects via M2 (β = −.03, BootSE = .03, 95% CI [−.09, .04]), M4 (β = −.01, BootSE = .03, 95% CI [−.06, .04]), and M5 (β = .01, BootSE = .03, 95% CI [−.05, .07]) were not significant and very small.

The results of the analysis in which the **RAGE** affective system was the independent variable (X) showed that this system significantly predicted the level of all schema domains (mediators) (**M1**: β = .30, p < .001; **M2**: β = .25, p < .001; **M3**: β = .32, p < .001; **M4**: β = .19, p < .001; **M5**: β = .15, p < .01). Among the mediators, only the Disconnection/Rejection domain (**M1**) was significantly associated with loneliness (**Y**) (β = .90, p < .001). The remaining domains were not significantly associated with loneliness. The **total effect** of the RAGE system on loneliness was significant and positive (β = .19, SE = .13, 95% CI [.19, .69]). However, after accounting for the mediators, the **direct effect** of this system on loneliness was nonsignificant (β = −.04, SE = .09, 95% CI [−.26, .09]), indicating **full mediation**. The **total indirect effect** was significant (β = .22, BootSE = .05, 95% CI [.13, .31]), with the only significant indirect effect occurring through the Disconnection/Rejection domain (**M1**) (β = .27, BootSE = .05, 95% CI [.17, .37]). The indirect effects via M2 (β = −.02, BootSE = .02, 95% CI [−.05, .01]), M3 (β = −.02, BootSE = .02, 95% CI [−.06, .01]), M4 (β = −.01, BootSE = .01, 95% CI [−.03, .02]), and M5 (β < .01, BootSE = .01, 95% CI [−.02, .02]) were not significant and very small.

In the next analyzed model, where the **PLAY** system (**X**) was the independent variable, this variable was significantly and negatively associated with four of the five schema domains (**M1**: β = −.35, p < .001; **M2**: β = −.28, p < .001; **M4**: β = −.19, p < .001; **M5**: β = −.38, p < .001). The Impaired Limits domain (**M3**) was not significantly associated with PLAY (β = −.04, p > .05). Among the mediators, only the Disconnection/Rejection domain (**M1**) was a significant predictor of loneliness (Y) (β = .84, p < .001). The remaining domains were not significantly associated with the sense of loneliness. The **total effect** of the PLAY system on loneliness was significant and negative (β = −.43, SE = .13, 95% CI [–1.42, −.89]), indicating that activation of the PLAY system is associated with a lower level of loneliness. After accounting for the mediators, the **direct effect** of PLAY also remained significant (β = −.19, SE = .10, 95% CI [−.70, −.31]), although its magnitude decreased, suggesting partial mediation. The total indirect effect was significant (β = −.24, BootSE = .04, 95% CI [−.32, −.16]), with the **only significant mediator** being the Disconnection/Rejection domain (**M1**) (β = −.29, BootSE = .05, 95% CI [−.38, −.20]). The remaining indirect effects via M2 (β = .02, BootSE = .02, 95% CI [−.01, .06]), M3 (β < .01, BootSE < .01, 95% CI [−.01, .01]), M4 (β < .01, BootSE = .01, 95% CI [−.02, .03]), and M5 (β = .02, BootSE = .02, 95% CI [−.03, .07]) were very small and did not reach statistical significance.

## Discussion

From a biological perspective, human personality – including the maladaptive cognitive schemas that form its framework – is determined by the functioning of extensive neural connection networks [20]. Based on the neurodevelopmental theory of depression [51], three key periods can be distinguished in the development of the biological foundation of personality: the prenatal stage, early childhood (up to 5–6 years of age), and adolescence. These same developmental windows are crucial for the proper maturation of the limbic system, the prefrontal cortex, and the network of interconnections between them [52]. The maturation stages of these structures also appear to coincide with the critical moments for external environmental influences that condition the emergence of early maladaptive schemas. The quality of developmental processes at these stages determines an individual’s functioning in the areas of motivation, adaptive processes, and self-experience in relationships with others [53]. The fundamental emotional systems described within affective neuroscience represent evolutionarily evolved subcortical neural networks that organize emotional, motivational, and behavioral responses. In particular, the SEEKING, FEAR, RAGE, CARE, PLAY, and SADNESS / PANIC / SEPARATION DISTRESS systems play a crucial role in regulating social behavior and responses to relational threats [19]. It should be noted that the BANPS assesses self-reported personality traits corresponding to primary emotional systems proposed by affective neuroscience theory and does not provide a direct measure of neural activity.

As discussed earlier, the role of affective systems is to regulate complex information-processing and behavioral control functions essential for survival [18], including the capacity to form satisfying social relationships. In line with Żechowski’s [54] proposition, instinctive affective processes – as a form of “evolutionary memory of mammals” – constitute the first level of information processing. The second level consists of cognitive processes based on learning (including learned coping strategies), while the third comprises self-awareness, expressed through reflectiveness, emotional regulation, behavioral control, adaptability to environmental demands, cognitive flexibility, and mentalization. Young’s early maladaptive schemas – together with the coping strategies that manifest in behavior – can be found on the latter two levels. However, this does not imply a lack of connection to the lowest, affective-processing level. The relationship between these processes is essential – they are not isolated functions, but layers of processing within the same psychological mechanism. Higher-level processes enable the integration of cognitive and affective elements, making them accessible to reflection and insight [54]. From the perspective of Young’s theory, early relational experiences lead to the development of relatively enduring cognitive-emotional structures referred to as early maladaptive schemas [35]. The present study hypothesized that individual differences in the reactivity of basic emotional systems may be related to susceptibility to the development of specific schema domains in response to specific early childhood experiences.

Our correlation analysis results concerning the relationships between affective systems and early maladaptive schemas (Table 3) are consistent with findings from our previous research [38,39]. The strongest associations with both schema domains and individual schemas were found for four systems: SADNESS / PANIC / SEPARATION DISTRESS, FEAR, RAGE, and PLAY (negative correlations in the latter case). This suggests that the activation of systems linked to intense experiences of separation anxiety, fear, and anger is accompanied by the formation and functioning of early maladaptive schemas, supporting their biological basis. An interesting observation is the consistently smaller and less significant relationships between SEEKING and CARE systems and early maladaptive schemas. For the SEEKING system, this result may be explained by Panksepp’s description of its deactivation in response to painful relational rejection experiences [27]. The lack of a significant association between the SEEKING system and loneliness may be due to the measurement characteristics of this variable. Within Panksepp’s framework, SEEKING is conceptualized as a motivational system related to exploration, anticipation, curiosity, and goal-directed engagement with the environment. However, the BANPS operationalization of SEEKING may capture broader exploratory and cognitive tendencies rather than specifically social motivation or interpersonal connectedness. Consequently, high SEEKING activation may not necessarily buffer against loneliness, particularly when exploratory tendencies are directed toward non-social goals or cognitive stimulation rather than relational engagement. This measurement-related specificity may partly explain why SEEKING was not significantly associated with loneliness in the present study. Future studies could further differentiate between social and non-social aspects of exploratory motivation when examining the relationship between primal emotional systems and loneliness.

The less pronounced link for the CARE system may be understood in the context of relational difficulties observed in adulthood that may be associated with early maladaptive schema activation – an individual with a hyperactive SADNESS / PANIC / SEPARATION DISTRESS system in childhood is likely to experience difficulties in both forming and maintaining secure social relationships and may exhibit deficits in expressed concern or caregiving towards others [55,56].

Our analyses also demonstrated that all early maladaptive schemas and their domains are significantly and positively associated with loneliness (Table 3). Among the primal emotional systems, significant positive correlations with loneliness were found for the SADNESS / PANIC / SEPARATION DISTRESS, FEAR, and RAGE systems. Negative correlations were observed for the CARE and PLAY systems, indicating that the activation of systems responsible for seeking and maintaining close social relationships can play a role in protecting against excessive loneliness (Table 3). It should be noted that, once again, we did not obtain the expected statistical confirmation of the relationship between the SEEKING system and the intensity of loneliness. One explanation may once more lie in the deactivation of this system when satisfying social experiences are lacking. These results are consistent with numerous studies conducted by Panksepp’s team [27,31] and other researchers (e.g., Vitale & Smith [25]). However, alternative explanations are possible. The SEEKING system is activated not only in social situations but may also manifest in non-social forms of activity (as the items in this scale relate to cognitive curiosity associated with problem-solving or creative thinking). Therefore, the lack of significant relationships between loneliness and this system may reflect the relatively low saturation of socially oriented items in the BANPS SEEKING subscale, which in turn may explain its weak links to patterns of forming interpersonal relationships.

When analyzing the mediating influence of early maladaptive schema domains (Table 4) between individual affective systems and loneliness, we found in most cases a significant mediating role of the Disconnection/Rejection domain between the activity of the SADNESS / PANIC / SEPARATION DISTRESS, FEAR, and RAGE systems (positive effect) and the CARE and PLAY systems (negative effect) and the level of loneliness. Thus, early childhood social experiences associated with the absence of the primary need for belonging, love, and relational security amplify the biologically rooted association of emotional system activation, making the experience of loneliness more intense (and conversely – positive early relational experiences may be associated with lower levels of loneliness). In addition, the Impaired Limits domain showed a positive mediating relationship between the FEAR system and loneliness. This domain results from the unmet need for realistic boundaries, which is often also linked to insufficient attentiveness to the developing child. Again, a lack of sufficient care and attention in critical developmental periods (the aforementioned early childhood and adolescence) may be associated with heightened anxiety sensitivity, which may co-occur with social distancing, fear of rejection, and greater loneliness. Moreover, heightened reactivity to negative social stimuli (e.g., criticism, lack of acceptance) may be associated with avoidance of interactions and reinforce isolation. It is also often linked to a tendency toward rumination and the experience of internal tension, both of which may further hinder the formation of social connections. One another possible explanation is that individuals characterized by externally oriented or impulsive behavioral styles (impaired limits) may engage more frequently in social activity despite experiencing fear, which could buffer subjective loneliness. However, this interpretation is speculative and requires further empirical investigation.

Although the findings we present are the first to address the topic in this specific way, they are consistent with the theoretical assumptions of early maladaptive schema theory [34] and affective neuroscience [27]. Our hypothesis, proposing the dominant role of the Disconnection/Rejection domain as a mediator between biological factors and loneliness, was confirmed.

The present findings may have several implications for psychological assessment and intervention. First, the observed associations between primary emotional systems and early maladaptive schemas suggest that clinicians working with individuals experiencing loneliness may benefit from assessing both affective temperamental tendencies (e.g., heightened SADNESS / PANIC / SEPARATION DISTRESS or FEAR) and schema-related cognitive–emotional patterns. Second, the prominent role of the Disconnection/Rejection schema domain in the statistical models indicates that therapeutic approaches focusing on unmet attachment needs and maladaptive relational expectations – such as schema therapy or other attachment-oriented interventions – may be particularly relevant for individuals reporting chronic loneliness. Third, the negative associations observed for CARE and PLAY systems may suggest that interventions strengthening prosocial engagement, emotional closeness, and playful social interaction could be useful components of preventive or therapeutic programs (e.g., imaginal work on corrective emotional experiences and a strong therapeutic alliance in the form of limited reparenting).

Given the small number of studies on this subject, it is important to highlight the innovative nature of our results and to encourage further exploration of these issues. The nature of the findings is specific to the cultural and demographic context of Poland, meaning that they may not be fully generalizable to other cultures. Another limitation is the relatively young age of the participants (*M* = 26.9; *SD* = 10.7), despite the relatively large sample size, which limits the generalizability of the results to older populations or clinical populations. Future studies should replicate the present findings in more diverse and clinically representative samples.

Another limitation concerns the characteristics of the study sample and the recruitment procedure. Participants were recruited online via social media platforms.

An additional limitation concerns the assessment of psychiatric history. Although participants reporting a history of psychiatric disorders were excluded from the study, this information was obtained through self-report rather than a structured clinical interview. Consequently, the presence of unreported, undiagnosed, or inaccurately reported psychiatric conditions cannot be ruled out, which may have influenced the observed associations.

For future research directions, it should be emphasized that emotional systems, in addition to their anatomical basis, have neurophysiological foundations – their functioning depends on the activity of specific neurotransmitters and neuromodulators (e.g., gamma-aminobutyric acid, dopamine, oxytocin, noradrenaline, serotonin) – which opens avenues for further analysis.

It should be emphasized that the mediation analyses used in the study represent statistical models rather than causal processes, therefore longitudinal or experimental studies would be necessary to test causal pathways.

Future studies should employ pre-registration and longitudinal designs.

The observed pattern of correlations suggests that higher scores on systems related to SADNESS / PANIC / SEPARATION DISTRESS, FEAR, and RAGE tend to co-occur with greater endorsement of early maladaptive schemas. However, the cross-sectional design of the present study does not allow conclusions about causal relationships between these variables.

Our findings should be interpreted as statistical associations rather than causal effects. It is also possible that individuals who already experience higher levels of loneliness may retrospectively report greater maladaptive schemas, or that other variables (e.g., personality traits or current relational context) contribute simultaneously to both schema activation and loneliness. Positive early relational experiences may therefore be related to lower levels of loneliness, although longitudinal studies would be required to determine the direction of this relationship.

A further limitation of this study is its cross-sectional design, which precludes any conclusions regarding causal relationships between the investigated variables. Although mediation analyses were conducted, the observed indirect effects should be interpreted as statistical associations consistent with the proposed theoretical model rather than evidence of causal pathways or underlying mechanisms. Longitudinal and experimental studies are needed to examine the temporal and potentially causal relationships among these constructs.

An additional limitation concerns the relatively low internal consistency of the CARE subscale of the BANPS (Cronbach’s α = 0.53). This level of reliability indicates a substantial amount of measurement error, which may have attenuated or distorted associations involving this dimension. Consequently, findings related to CARE should be interpreted with particular caution and regarded as preliminary. Further studies using measures with stronger psychometric properties are needed to verify the robustness of these associations.

Another limitation of the present study concerns the use of self-report measures to assess loneliness, emotional systems, and early maladaptive schemas. Self-report instruments are inherently susceptible to several sources of bias, including social desirability, recall bias, and individual differences in self-awareness.

## Conclusions

1. Early childhood relational experiences related to unmet needs for belonging, love, and relational security were associated with stronger relationships between primal emotional systems and loneliness.
2. The Disconnection/Rejection schema domain emerged as the most consistent statistical mediator in the associations between several primal emotional systems and loneliness.
3. Due to the cross-sectional and self-report nature of the study, the findings should be interpreted as associations rather than evidence of causal relationships.
